# Recent Patents of Interest to Dentists

**Published:** 1907-01

**Authors:** 


### RECENT PATENTS OF INTEREST TO DENTISTS.

836299. Dentists' tweezers, Welby H. Burgin, Richmond, Ky.
836967. Dental mirror, Walter H. Grant, Boston, Mass.
837871. Tooth-brush and dentifrice bracket, Lewis W. McConnell and W. W. Gage, McCook, Nebr.
837423. Dental handpiece, Alson C. Sargent, Des Moines, Iowa.
838296. Dental work, Harrison D. Best, Pittsburg, Pa.
838299. Head-rest, Arthur W. Browne, Prince Bay, N. Y.
838027. Tool for handling artificial teeth, Frederick L. Hunt, Asheville, N. C.
838415. Artificial tooth, Jonh H. Jackson, Boston, Mass.
838849. Porcelain and metallic crown, Charles A. Davis, Pa sadena, Cal.
838648. Combination hand mouth-mirror and chip blower and flushing and spraying device, Oliver T. Robertson, Milford, O.

Copies of the above patents may be obtained for fifteen cents each, by addressing John A. Saul, Solicitor of Patents, Fendal Building, Washington, D. C.

